# CXCR3 Antagonism of SDF-1(5-67) Restores Trabecular Function and Prevents Retinal Neurodegeneration in a Rat Model of Ocular Hypertension

**DOI:** 10.1371/journal.pone.0037873

**Published:** 2012-06-04

**Authors:** Alexandre Denoyer, David Godefroy, Isabelle Célérier, Julie Frugier, Julie Degardin, Jeffrey K. Harrison, Francoise Brignole-Baudouin, Serge Picaud, Francoise Baleux, José A. Sahel, William Rostène, Christophe Baudouin

**Affiliations:** 1 UPMC University Paris 6, Institut de la Vision, UMRS968, Paris, France; 2 INSERM, U968, Paris, France; 3 CNRS, U7210, Paris, France; 4 Quinze-Vingts National Ophthalmology Hospital, Paris, France; 5 Team 1, Centre de Recherche des Cordeliers, INSERM, U872, Paris, France; 6 Department of Pharmacology & Therapeutics, College of Medicine, University of Florida, Gainesville, Florida, United States of America; 7 Department of Toxicology, Faculty of Biological and Pharmacological Sciences, University René Descartes Paris 05, Paris, France; 8 Unité de chimie des biomolécules, Institut Pasteur, CNRS 2128, Paris, France; Massachusetts Eye & Ear Infirmary, Harvard Medical School, United States of America

## Abstract

Glaucoma, the most common cause of irreversible blindness, is a neuropathy commonly initiated by pathological ocular hypertension due to unknown mechanisms of trabecular meshwork degeneration. Current antiglaucoma therapy does not target the causal trabecular pathology, which may explain why treatment failure is often observed. Here we show that the chemokine CXCL12, its truncated form SDF-1(5-67), and the receptors CXCR4 and CXCR3 are expressed in human glaucomatous trabecular tissue and a human trabecular cell line. SDF-1(5-67) is produced under the control of matrix metallo-proteinases, TNF-α, and TGF-β2, factors known to be involved in glaucoma. CXCL12 protects *in vitro* trabecular cells from apoptotic death *via* CXCR4 whereas SDF-1(5-67) induces apoptosis through CXCR3 and caspase activation. Ocular administration of SDF-1(5-67) in the rat increases intraocular pressure. In contrast, administration of a selective CXCR3 antagonist in a rat model of ocular hypertension decreases intraocular pressure, prevents retinal neurodegeneration, and preserves visual function. The protective effect of CXCR3 antagonism is related to restoration of the trabecular function. These data demonstrate that proteolytic cleavage of CXCL12 is involved in trabecular pathophysiology, and that local administration of a selective CXCR3 antagonist may be a beneficial therapeutic strategy for treating ocular hypertension and subsequent retinal degeneration.

## Introduction

Primary open-angle glaucoma affects about 70 million people and is predicted to account for over 11 million cases of blindness by 2020 [1], [2]. Its prevalence continues to increase as the population ages. Glaucoma is a retinal neuropathy characterized by retinal ganglion cell death. Pathological elevation of intraocular pressure (IOP), namely ocular hypertension (OHT), is the most critical risk factor for both the development and the progression of the disease [3]. OHT is often diagnosed several years before detecting the neuropathy. It is attributed to a decrease in trabecular meshwork (TM) outflow facility to aqueous humor (AH) caused by tissue degeneration whose primary mechanisms are still unclear. Classical antiglaucoma treatments reduce the abnormally elevated IOP but do not target directly the initial TM pathology. In clinical practice, progressive therapeutic inefficiency in controlling both the elevation of IOP and neuropathy often occurs [4]. The lack of specific therapies for the TM pathology, which is still developing in well-treated patients, could be responsible for progressive treatment inefficiency coupled with neuropathy worsening and sometimes blindness.

TM degeneration has largely been demonstrated as the main cause of aqueous outflow resistance leading to OHT in primary open-angle glaucoma (5]. The main glaucoma-related trabecular modifications resemble age-related TM degeneration and involve accumulation of trabecular extracellular matrix together with a decrease in TM cellularity as previously described by our group and others [6]–[9]. Trabecular cell (TC) loss that occurs in glaucoma is known to develop through apoptotic phenomena and was found as a characteristic of primary open-angle glaucoma [10], but its causal mechanisms are still unknown.

Stromal cell-derived factor-1 (SDF-1), termed CXCL12, belongs to the CXC subfamily of chemokines. CXCL12 is known to bind mainly to a G-protein coupled receptor, CXCR4. Recently, CXCR7 has been identified as an additional receptor for CXCL12 [11]–[13]. Interestingly, CXCL12 is not only involved in the immune system, but also in axonal development and neurotransmission [14], [15], migration, proliferation, and survival of cancer cells [16], and extracellular matrix adhesion of haematopoietic cells in bone marrow or damaged tissues [17], [18]. In the eye, CXCL12 and CXCR4 have been hypothesized to play a role in neovascularization and in ocular inflammation since they were detected in the retina [19], [20], the cornea [21], and the AH [22]. Matrix metalloproteinase (MMP) proteolysis is one of the regulating factors for chemokine activity [23], [24]. Proteolytic processing of CXCL12 yields a wide variety of amino-terminal truncated proteins that lose their ability to bind to CXCR4 [25] as this chemokine–receptor interaction requires the CXCL12 N-terminal residues [26]. One of the cleaved forms of CXCL12, SDF-1(5-67), has been reported to induce neuronal apoptosis during HIV brain infection [27]. Recently, SDF-1(5-67) has been shown to bind specifically to another chemokine receptor, CXCR3, where it induces direct neuronal apoptosis [28].

In the present study, highly selective non-peptide antagonists of CXCR3 and CXCR4 were studied for their effects on OHT and related retinal neurodegeneration. We show that ocular administration of a CXCR3 antagonist lowers IOP, prevents retinal ganglion cell degeneration, and protects visual function in an animal model of OHT. The chemokine and both receptors were detected in human glaucomatous trabecular tissue and a trabecular cell line. SDF-1(5-67) was found to be produced by trabecular cells under the control of MMPs and cytokines known to be involved in glaucoma. We demonstrate that SDF-1(5-67) induces TC apoptosis through CXCR3, and that blocking CXCR3 restores the filtrating function of the TM and protects the retina against OHT-related degeneration. Collectively, the results suggest that pathological enhancement of a SDF-1(5-67)/CXCR3 interaction is involved in trabecular degeneration and this chemokine/chemokine receptor axis may represent a new therapeutic target to prevent the deleterious effect of OHT on the visual function.

## Methods

### Reagents

Recombinant human CXCL12 (7.8 kDa), TNF-α (17.5 kDa), TGF-β2 (12.8 kDa), CXCL10 (8.7 kDa) and TIMP-1 (20.6 kDa) were obtained from R&D Systems (Minneapolis, MN, USA). CXCL11 (8.3 kDa) was obtained from Preprotech (Rocky Hill, NJ, USA). Exogenous CXCL12 and truncated SDF-1(5-67) (7.4 kDa) were synthesized by F. Baleux. NBI-74330, a non-peptide antagonist of CXCR3 [29], [30], and AMD-3100, a non-peptide antagonist of CXCR4 [31], were synthesized by Orga-Link (Gif-sur-Yvette, France). Batimastat was a gift from E. Gabison and S. Menashi (CNRS UMR 7149, University of Paris 12, Créteil, France). Cell antigens (Ag) were detected in TCs using the following antibodies (Ab): mouse IgG1 anti-human CXCL12 mAb (1∶200, clone 79018, R&D Systems), mouse IgG2B anti-human CXCR4 mAb (1∶200, clone 44716, R&D Systems), mouse IgG1 anti-human CXCR3 mAb (1∶200, clone 49801, R&D Systems), anti-caspase 3 (clone 92-605, BD Biosciences), secondary antibodies Alexa Fluor488 goat anti-mouse IgG (1∶500, Molecular Probes, Montluçon, France), and FITC-conjugated goat anti-mouse Fab (1∶500, DakoCytomation, Glostrup, Denmark). Rat TM were assessed for inflammatory cell infiltration using primary rabbit anti-CD45 pAb (1∶100, ab10558. Abcam, Cambridge, MA, USA) and mouse anti-rat CD11b mAb (1∶200, ab8879, Abcam). Anti-SDF-1(5-67) (1∶100) was a generous gift from C. Overall (University of British Columbia, Vancouver, Canada), and anti-CXCR7 (1∶200) was given by M. Thelen (Institute for Research in Biomedicine, Bellinzona, Switzerland). Isotype-matched antibodies from R&D Systems were used as negative controls.

### Human Trabecular Meshwork Specimens

Human TMs were obtained from 15 patients undergoing surgery for primary open-angle glaucoma. All patients included in the study were diagnosed for glaucoma at least 1 year before and presented no other ocular pathology or systemic disease. The TM was selectively removed during non-penetrating deep sclerectomy. Experiments were conducted in the Clinical Investigation Centre for Ocular Surface Pathology (Centre Hospitalier National d’Ophtalmologie des Quinze-Vingts, Paris, France) in accordance with the Declaration of Helsinki, Scotland amendment, 2000. National ethics committee approval was obtained (INSERM-DHOS CIC, 503) and all patients signed the informed consent form before surgery.

### Human Glaucomatous Trabecular Cell Line

For *in vitro* experiments, the human glaucomatous TC line HTM3 was used, which has been previously characterized [32]. HTM3 cells were routinely cultured in standard humidified 5% CO_2_ atmosphere in serum-free Dulbecco’s Modified Eagle Medium (DMEM. GIBCO, Invitrogen, Carlsbad, CA, USA) supplemented with 4 mM L-glutamine, 10% fetal bovine serum, and 50 µg/mL gentamicin. For passages, monolayers were rinsed with PBS, dislodged by trypsinization (0.25% trypsin, 0.02% EDTA), then cultured from an initial concentration of 60,000 cells/mL. For immunofluorescence, TCs were grown on 22-mm glass cover-slips, then dried and fixed in 4% paraformaldehyde for 15 min. All cells were used at passage 10 to 20.

### Animal Model

Male 8-week-old Long-Evans rats weighing 300–350 g were used. Animals were kept in pathogen-free conditions with food and water available *ad libitum* and housed in a 12-h light/12-h dark cycle. Ocular integrity was checked using the slit lamp biomicroscope. The surgical model of OHT was induced in the right eye of each rat by cauterization of three episcleral veins after conjunctival dissection under general anesthesia (intraperitoneal injection of ketamine 75 mg/kg and xylazine 10 mg/kg) as reported elsewhere [33]. The left eyes underwent conjunctival dissection only as controls. After the surgery, the animals were maintained for a 21-day period and monitored for IOP three times a week using a handheld tonometer (TonoLab, Medtronics, Jacksonville, FL, USA) without sedation. Animals presenting low or instable IOP were then excluded, and the experiments were carried out at least one month after the surgery. Institutional review board approved the animal ethics for this study. All experiments were conducted in accordance with the Association for Research in Vision and Ophthalmology for the Use of Animals in Ophthalmic research.

### Real-time PCR

mRNA levels in human tissues and TC line were assessed using RT 7300 (Applied Biosystems) and assays-on-demand primers for human CXCL12 (ID Hs00171022m1), human CXCR4 (ID Hs00607978s1), and human CXCR3 (ID Hs00171041m1). Total mRNA was isolated from cultured TCs using the NucleoSpin RNA II extraction kit (Macherey-Nagel, Düren, Germany), and was then reverse-transcribed (TaqMan Reverse Transcription Reagents, Applied Biosystems). mRNA in human TM tissue was extracted using the NucleoSpin RNA XS kit (Macherey-Nagel), then conditioned and determined following the same above-described procedure. Relative quantitation of target genes was calculated according to the comparative Ct method, *i.e.* normalized to an endogenous control S18 gene and relative to a calibrator after calculating the efficiency coefficient. The results are presented as the inverse of the normalized Ct value (InvCt) or as the relative fold change compared to unstimulated control. A negative control was routinely used omitting mRNA from the RT reaction mixture.

### Immunohistofluorescence

Protein expression of CXCR3, CXCR4, and CXCL12 was detected in tissues and in the HTM3 cell line by indirect immuno-fluorescence using a laser confocal microscope (E800, PCM2000, Nikon, Champigny-sur-Marne, France). Nonspecific Ab binding was blocked with normal goat serum or with fetal bovine serum in PBS/0.1% Triton for 60 min, washed three times, then incubated overnight at 4°C with primary Ab. After three more washes, cells were counterstained with secondary fluorescent Ab for 2 h at room temperature. Specimens were finally washed three times in PBS, incubated in propidium iodide for 3 min (Sigma-Aldrich, France) or in DAPI for 1 min, and mounted in gel mounting medium. Isotype-matched mAbs were used as controls for each sample and each primary Ab.

### Flow Cytometry

Total and cell membrane surface expression of proteins was analyzed on a FC 500 flow cytometer (Beckman Coulter, Miami, FL, USA). TCs (120,000 per well) were plated in six-well plates (Costar, Cambridge, MA, USA) and grown to 80% confluence. The cells were cultured in complete medium for 24 h and subsequently incubated with serum-free DMEM, supplemented CXCL12, or SDF-1(5-67) for 12 h. Cells were carefully detached, suspended in PBS containing 1% BSA, blocked with normal rabbit serum, and stained with primary Ab for 1 h on ice, washed three times with cold 1% BSA PSB, followed by counterstaining with the corresponding fluorescein isothiocyanate-conjugated secondary antibody (DakoCytomation) for 30 min. Cells were preincubated and immunostained adding or not 0.5 mg/mL saponin in order to determine total or surface expression respectively. Isotype-matched antibodies were used as negative controls. For each specimen, at least 1,000 cells per antibody were analyzed. The results are reported as mean fluorescence intensity (MFI) normalized to isotypic control.

### ELISA

The amount of CXCL12 released into TC supernatants was measured by an in-house ELISA kit (ref. DY350, R&D Systems). Cells were cultured and stimulated as described above, and supernatants were isolated after centrifugation. 96-well microtitre plates were coated overnight with mouse anti-CXCL12 capture mAb then nonspecific binding was blocked for 2 h with 1% BSA in PBS. Duplicate samples (100 µL) of cell supernatants or serial dilutions of standards of human recombinant CXCL12 were incubated for 2 h, washed, then incubated with goat anti-CXCL12 mAb for 2 h, followed by 20-min incubation with HRP-conjugated streptavidin. Reaction product was detected using color reagent (ref. DY999, R&D Systems). Optical density was read (450–570 nm, SpectraFluor, TECAN, Switzerland) and averaged from ten measurements per well. The limit of sensitivity for this assay was 15 pg/mL.

### Western Blot Analysis

Protein extracts were prepared from TCs (120,000 cells per well) grown to 80% confluence in six-well plates. The cells were maintained with medium for 24 h, then incubated subsequently for 24 h with supplemented DMEM only or with specific inhibitors, detached and homogenized in cell lysis buffer. Equal amounts of proteins were separated on 4%–12% Tris-Glycine gel (Novex, Invitrogen), transferred to nitrocellulose, and probed overnight with anti-SDF-1(5-67) neoepitope polyclonal antibody (1∶100, 30-min preincubated with CXCL12 to improve the antibody specificity) following carefully the same procedure as previously described [28]. Primary Ab binding was revealed using HRP-conjugated goat anti-rabbit IgG (1∶3000, Vector, AbCys, Paris, France) for 1 hour and developed with ECL Plus detection reagents (GE healthcare, Orsay, France). β-actin (Abcam) was used as internal control. Signal intensity of anti-SDF-1(5-67) binding was quantitated using ImageJ software and normalized to β-actin detection. Recombinant CXCL12 and SDF-1(5-67) were used in Western Blots in order to control the antibody for specificity against SDF-1(5-67) neoepitope only.

### TUNEL Labeling

A terminal deoxynucleotidyl transferase-mediated dUTP nick-end labeling (TUNEL) assay (Roche Diagnostics, Meylan, France) was performed to detect apoptosis in rat eye cryosections following the manufacturer’s instructions. Controls were done in each eye using a 10-min pretreatment with DNAse or omitting the transferase solution as positive or negative control, respectively. Specimens were mounted in aqueous mounting medium with DAPI to be further analyzed using light epifluorescence microscopy. TUNEL-labeled TCs were observed and counted in five 0.01 mm^2^ fields per sample in order to compare apoptotic TC density between groups. TUNEL-labeled retinal ganglion cells were counted in five fields per sample and normalized to the observed retinal inner layer length.

### Cell Viability Assays

The effects of CXCL12 and SDF-1(5-67) on the cell proliferation and apoptosis were analyzed in a previously used cellular model of toxicity [34]: 80% confluent TCs were incubated in PBS with 0.01% benzalkonium chloride for 15 min in order to induce approximately 50% apoptosis. Cells were washed with PBS, incubated in free medium (control) or with CXCL12, SDF-1(5-67), and inhibitors during a 24-h recovery period. Microplate cytofluorometry was performed on Saphire Microplate reader (Tecan Instruments, Lyon, France) in 96-well microtitre plates: neutral red staining (Fluka, Ronkonkoma, NY, USA) was used to evaluate membrane integrity that closely correlates with cell viability [35]; apoptosis was quantified with the nuclear dye Hoechst33258 (Hoechst, Germany) combined with propidium iodide in order to exclude necrosis [36]. Apoptosis was calculated as the ratio of apoptotic cells on the total cell viability, and expressed as relative fold changes compared to control.

### Animal Experiments

For evaluation of the effect of non-peptide antagonists on IOP, forty animals presenting stable OHT were included and randomly divided into four equal groups: in both eyes, ten rats received one subconjunctival injection of the CXCR4 antagonist AMD3100 (1 µM, 100 µL), ten received a single injection of the CXCR3 antagonist NBI-74330 (1 µM, 100 µL), ten received one injection of NBI-74330 followed by a second one 4 days after, and ten rats received the vehicle only. IOP was monitored every two days by an independent person *i.e.* who was blind to the treatment. At the end of the experiments, animals were euthanized and the eyes were immediately removed, fixed in 4% paraformaldehyde, embedded in an optimal cutting-temperature compound (OCT, Tissue-Tek, Miles Inc, Bayer Diagnostic, Püteaux, France) and cut into 15-µm cryosections. For the NBI-74330 dose-response study, twenty animals presenting stable OHT were randomly divided into four equal groups to receive either NBI-74330 injections (100 µL) at three different concentrations (0.01 µM, 0.1 µM, and 1 µM) or the vehicle only. IOPs were averaged from days 6, 8, and 10.

For the evaluation of AH outflow, twenty animals presenting stable OHT were randomly divided into two equal groups to receive either NBI-74330 injections (1 µM, 100 µL) or the vehicle only. AH outflow was assessed at day 8 using *in vivo* fluorophotometry [37], [38]. Briefly, 1 µL fluorescein was injected using a 33G needle into the anterior chamber under general anesthesia. One hour after, AH decrease in anterior chamber fluorescence has been recorded *in vivo* for 30 minutes using Micron III fluorescent imaging device (Phoenix research laboratories, San Ramon, CA) and ImageJ software (NIH). Mean fluorescence intensities were normalized to the initial intensity and drawn in semi-log graphs as a function of time in order to calculate the AH outflow depending on the curve slope. To label the hydrodynamic patterns of trabecular outflow passage, ocular perfusion of fluorescent microsphere was performed as reported elsewhere [39], [40]. Ocular anterior chamber was exchanged with red-fluorescent microspheres in BSS (0.1 µm, 0.02%v/v, Invitrogen) using a double 30G needle infusion, and followed by a 30-minute perfusion at the IOP measured before death. Anterior chamber contents were washed with BSS and eyes were immediately removed and fixed in 4% paraformaldehyde for 15 minutes. Anterior segments were quadrisected and flat-mounted on coverglass after exposing the TM by gently moving the iris tissue. TM was observed under laser confocal microscope using a 20× objective lens regardless of the presence of microsphere accumulation. An index of the trabecular filtration, namely trabecular percent effective filtration length (PEFL), was calculated as the length ratio of the zones wherein tracer accumulated to the total length of the inner wall in at least 8 images per eye [39].

For the *in vivo* evaluation of retinal atrophy and visual function, twenty animals with stable OHT were injected in one eye with either NBI-74330 (1 µM, 100 µL) or vehicle (100 µM) following the same protocol as described above. IOP was monitored once a week. Retinal nerve fiber density was assessed *in vivo* 2 months after the treatment using confocal scanning laser ophthalmoscopy (HRA, Heidelberg Engineering, Heidelberg, Germany). The eyes were dilated with 0.5% tropicamide and 0.5% phenylephrine hydrochloride eye drops (Santen, Osaka, Japan). Each rat was gently held manually to keep the eye in position for imaging the retina. Dynamic retinal images were recorded in the center of the fundus and in the four mildperipheral areas. The density of retinal nerve fiber was calculated in each image and averaged using Metamorph software (Molecular Device, Sunnyval, CA, USA). Visual function was evaluated using an optokinetic apparatus consisting of interchangeable circular drums with black and white stripes rotating around a stationary holder in which the rat sits, as previously described [41]. Rats were tested at spatial frequencies of 0.125, 0.25, and 0.5 cycles/degree (2 turns/minute) under photopic conditions. Each rat was tested for one minute for each eye and each frequency during one session. Digital recordings of the head movements were analyzed and the amount of time the rat spent head tracking was calculated separately for each eye in a masked manner.

To study the effect of the chemokine on IOP, intraocular injections with CXCL12 or SDF-1(5-67) (100 ng/mL [13 nM], 5 µL) or vehicle only were performed in ten normal rats. The IOP was measured every 12 hours using Tonolab tonometer during five days.

### Statistical Analysis

All data in text and in bar graphs are reported as means ± SEM and represent at least three independent experiments. Analysis was performed using NCSS software (NCSS, Kaysville, UT, USA). Data were tested for distribution in order to perform the adequate parametric (Student’s t-test or one-way ANOVA followed by Tukey’s post hoc test) or nonparametric test (Mann-Whitney or Kruskal-Wallis tests) for the comparisons. A value of *P*<0.05 was considered to be significant.

## Results

### Human Glaucomatous Trabecular Tissue and A Trabecular Cell Line Express CXCL12, SDF-1(5-67), CXCR3 and CXCR4

**Figure 1:**
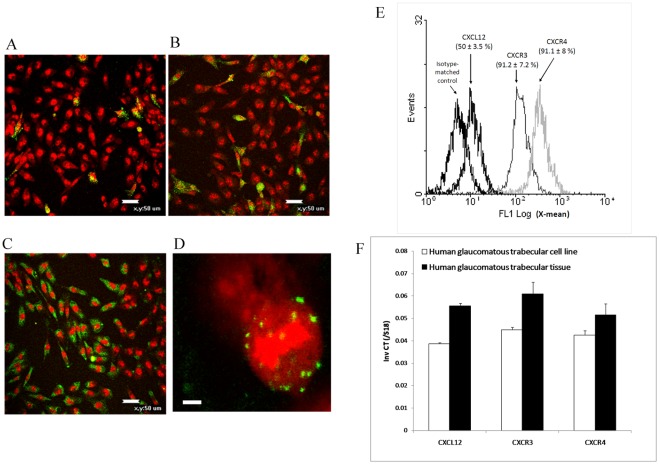
CXCL12, CXCR3, and CXCR4 expression by human glaucomatous trabecular tissue and a trabecular cell line. *(A–C)* The chemokine CXCL12 *(A)* and receptors CXCR3 *(B)* and CXCR4 *(C)* are detected in unstimulated human glaucomatous trabecular cells HTM3 by indirect immunofluorescence (secondary antibody in green, propidium iodide in red, scale bar: 50 µm, magnification ×200). *(D)* Chemokine receptor CXCR4 appears as distinct spots located at the cell membrane surface (scale bar: 5 µm, mag. ×800). Representative images of three independent experiments are depicted. *(D)* Cell expression of CXCL12 and receptors is also detected and quantified by immunoflowcytometry. Representative results obtained over 6 independent experiments, mean ± SEM of positive cells. *(E)* Chemokine and receptor mRNAs are detected in human glaucomatous trabecular tissues (n = 15) and in the HTM3 trabecular cell line. Data in the bar graph are presented as means ± SEM.

**Figure 2:**
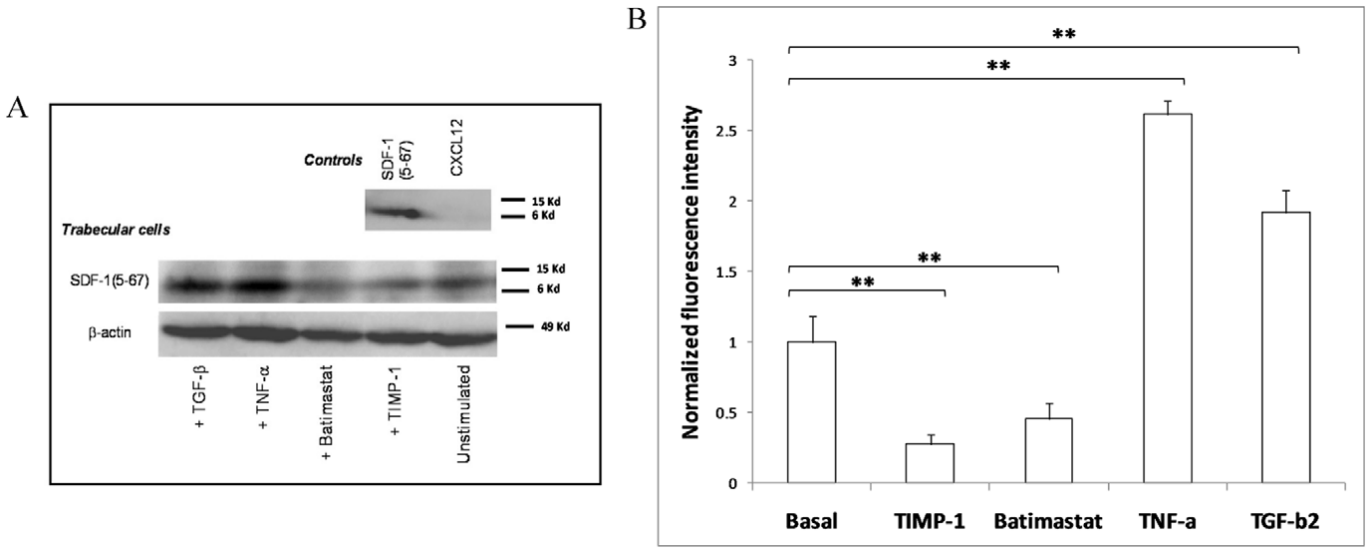
Human glaucomatous trabecular cells produce SDF-1(5-67). *(A,B)* SDF-1(5-67), a truncated form of CXCL12, is detected in the human glaucomatous trabecular cell line HTM3 using a specific anti-SDF-1(5-67) neoepitope antibody. MMP inhibitors batimastat (100 nM) and TIMP-1 (0.5 nM) induce a decrease in SDF-1(5-67) production, whereas TNF-α (50 ng/mL [2.9 nM]) and TGF-β2 (10 ng/mL [0.8 nM]) enhance the production of SDF-1(5-67). ** *P*<0.01. Exogenous SDF-1(5-67) and CXCL12 were used as positive and negative controls respectively for the antibody specificity as presented in the upper membrane (controls) of a representative western blot *(A)* taken from three independent experiments. Data in bar graphs are presented as means ± SEM.

We first checked whether human glaucomatous trabecular tissue and cells were able to express the chemokine and chemokine receptors. CXCL12, CXCR3, and CXCR4 were detected by immunofluorescence and flow cytometry in unstimulated cells (**Fig. 1** ***A–E***). mRNAs for CXCL12, CXCR3 and CXCR4 were also detected in both human tissue and trabecular cell line (**Fig. 1** ***F***). Finally, TCs were able to release CXCL12 as determined by ELISA of cell supernatants (22.8±5.28 pg/ml [2.9±0.7 pM] from 100,000 cells/mL grown during 48 h). Western blot analysis of unstimulated HTM3 cells, using a polyclonal anti-SDF-1(5-67) neoepitope-specific antibody, identified SDF-1(5-67) (**Fig. 2** ***A,B***). Incubation (24-h) with MMP inhibitors batimastat (100 nM) or TIMP-1 (0.5 nM) significantly reduced the amount of SDF-1(5-67), confirming that it originated from MMP activity produced by TCs. 24-h incubation of TCs with either TNF-α (50 ng/mL [2.9 nM]) or TGF-β2 (10 ng/mL [0.8 nM]), both known to be involved in glaucoma, increased the production of SDF-1(5-67) (**Fig. 2** ***A,B***).

### CXCL12 Protects TCs from Apoptotic Stress Whereas SDF-1(5-67) Induces Apoptosis

**Figure 3:**
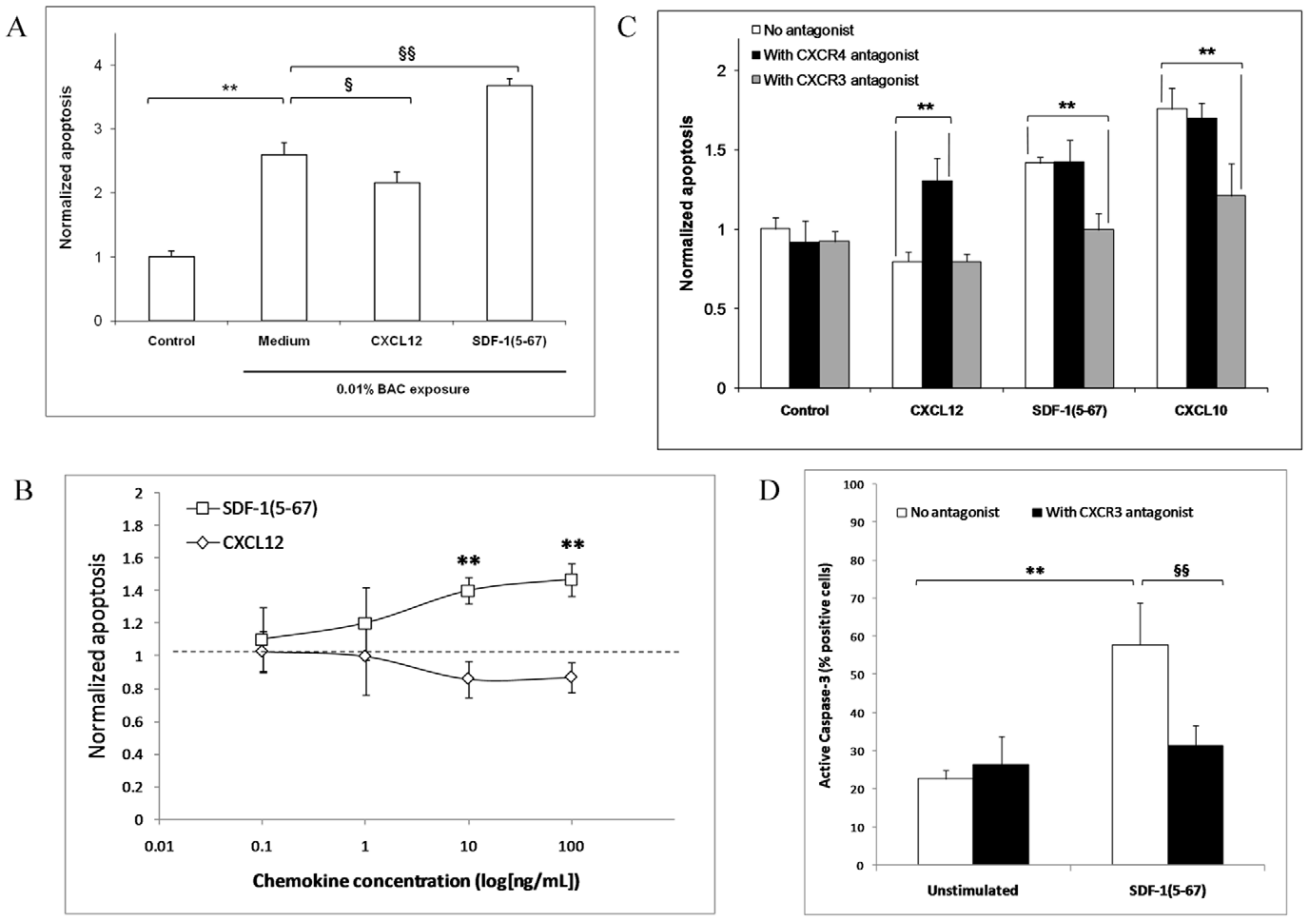
CXCL12 protects trabecular cells from apoptosis *via* CXCR4, whereas SDF-1(5-67) induces apoptosis through CXCR3 and caspase-3 activation. *(A)* 24-h incubation with CXCL12 (10 ng/mL [1.3 nM]) protects HTM3 cells from apoptotic stress induced by 15-min exposure to 0.01% benzalkonium chloride (BAC), whereas SDF-1(5-67) (10 ng/mL [1.3 nM]) increases apoptosis as assessed by microplate cytometry using Hoechst dye. ** *P*<0.05 vs. unstressed cells, § *P*<0.05 and §§ *P*<0.01 vs. BAC-exposed cells. *(B)* The protective effect of CXCL12 (10 ng/mL, 24 h) is reversed by CXCR4 antagonist (AMD-3100, 1 µM), whereas the apoptotic effect of SDF-1(5-67) (10 ng/mL, 24 h) is inhibited by CXCR3 antagonist (NBI-74330, 1 µM). CXCL10 (10 ng/mL [1.1 nM], 24 h), a conventional ligand for CXCR3, mimics the apoptotic effect of SDF-1(5-67). ** *P*<0.01. *(C)* Dose-dependent effect of 24-h incubation with SDF-1(5-67) or with CXCL12. ** *P*<0.01 vs. CXCL12. *(D)* SDF-1(5-67) (10 ng/mL) increases caspase-3 activation as assessed by immunoflowcytometry. CXCR3 antagonist (NBI-74330, 1 µM) inhibits SDF-1(5-67)-induced caspase 3 activation. ** *P*<0.01 vs. unstimulated, §§ *P*<0.01 vs. SDF-1(5-67)-stimulated. Data in graphs are presented as means ± SEM.

In a TC model of toxic-induced apoptosis [34], addition of CXCL12 (10 ng/mL [1.3 nM]) decreased apoptosis, whereas SDF-1(5-67) (10 ng/mL [1.3 nM]) potentiated apoptosis (**Fig. 3** ***A***). Both the protective effect of CXCL12 and the deleterious effect of SDF-1(5-67) were concentration-dependent, with a maximal effect of either chemokine observed at 10 ng/mL (**Fig. 3** ***B***).

### SDF-1(5-67) Induces Apoptosis via CXCR3 and Caspase-3 Activation

In order to test that CXCL12 and its cleaved form were acting *via* two different receptors, the effect of CXCL12/SDF-1(5-67) on receptor cell membrane expression was assessed using flow cytometry. We observed that exogenous CXCL12 (10 ng/mL [1.3 nM], 6 h) downregulated cell surface expression of CXCR4 but not CXCR3. In contrast, stimulation with SDF-1(5-67) reduced the membrane expression of CXCR3 but not CXCR4 (**Fig. S1**). TC expression of CXCR7, an additional CXCL12 receptor, was not affected by either CXCL12 or by SDF-1(5-67), (1±0.2, 1.2±0.1, and 0.95±0.05 for normalized mean fluorescence intensity in unstimulated, CXCL12- and SDF-1(5-67)-stimulated cells, respectively). Blockade of CXCR4 with AMD3100 (1 µM) inhibited the protective effect of CXCL12 and induced apoptosis, mimicking the apoptotic effect of SDF-1(5-67) (**Fig. 3** ***C***). I-TAC/CXCL11, a ligand for CXCR3 and CXCR7, had no effect on TC apoptosis (1.04±0.12, 1.11±0.10, and 1.03±0.14 fold over control for a 24-h incubation with CXCL11 at 1 ng/mL, 10 ng/mL, and 100 ng/mL respectively) confirming that the protective effect of CXCL12 was mediated by CXCR4. In contrast, selective blockade of CXCR3 with NBI-74330 (1 µM) inhibited the apoptotic effect of SDF-1(5-67) (10 ng/mL) (**Fig. 3** ***C***). Moreover, 24-h incubation with CXCL10 (10 ng/mL [1.1 nM]), another ligand for CXCR3, induced cell apoptosis that was prevented by NBI-74330. SDF-1(5-67) increased active caspase-3 and this effect was also inhibited by the CXCR3 antagonist (**Fig. 3** ***D***).

### In vivo Treatment with a CXCR3 Antagonist Reduces Ocular Hypertension in a Rat Model of Ocular Hypertension

**Figure 4:**
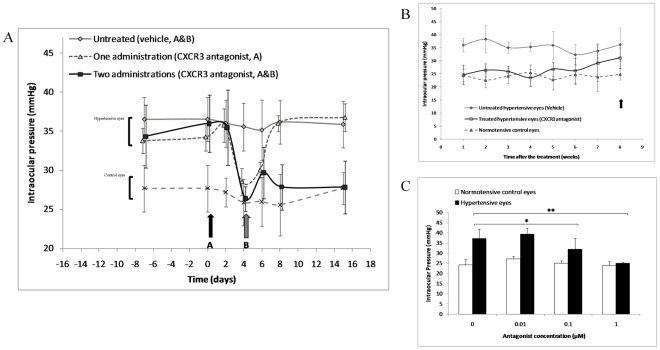
Ophthalmic administration of CXCR3 antagonist decreases intraocular pressure in a rat model of ocular hypertension. *(A)* A single administration of CXCR3 antagonist (NBI-74330, 1 µM, 100 µL) induces a transient decrease in intraocular pressure (n = 10 in each group). *(B)* When the antagonist is administrated twice, intraocular pressure remains low during 6 weeks (n = 10 each); the black arrow indicates the period of retinal and visual *in vivo* testing presented in *Fig. 6A,B*. *(C)* Dose-dependent effect of two administrations of CXCR3 antagonist on intraocular pressure as tested two weeks after the treatment (n = 5 each). * *P*<0.05, ** *P*<0.01. Data in graphs are presented as means ± SEM.

In order to extent our *in vitro* data, we tested *in vivo* whether CXCR3 and/or CXCR4 were implicated in the regulation of IOP by using highly selective non-peptide antagonists of both chemokine receptors in a rat model of OHT and related retinal degeneration, which was induced by episcleral vein cauterization [29]. AMD-3100 [31] (1 µM, 100 µL) or NBI-74330 [29], [30] (1 µM, 100 µL), selective antagonists for CXCR4 and CXCR3 respectively, were administrated in the subconjunctival space of rat eyes. A single administration of NBI-74330 induced a decrease in IOP 4 days after the treatment, reaching the normal IOP values observed in normotensive control eyes (**Fig. 4** ***A***). This decrease in IOP was transient since IOP returned to elevated values of untreated eyes after 6 days. When a second administration was given at the time of maximal decrease, IOP remained low during a period of 6 weeks (**Fig. *4B***). NBI-74330 reduced IOP in a dose-dependent manner (**Fig. 4** ***C***). In contrast, subconjunctival administration of a CXCR4 selective antagonist did not influence IOP in eyes with OHT (**Fig. S2**). In control eyes, the antagonists had no effect. In parallel, CXCL12 and SDF-1(5-67) were tested in normal rat eyes for their ability to modify IOP. CXCL12 ocular injections had no significant effect on IOP, whereas two injections of SDF-1(5-67) significantly increased IOP that remained elevated for 3 days (**Fig. S3**).

### CXCR3 Antagonist Improves Trabecular Function and Reduces Trabecular Cell Apoptosis

**Figure 5:**
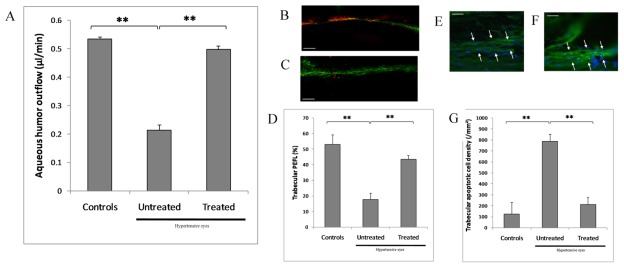
Ophthalmic administration of CXCR3 antagonist restores trabecular filtrating function and protects trabecular cells from apoptosis in a rat model of ocular hypertension. *(A)* Aqueous humor outflow impairment in hypertensive eyes is counteracted by treatment with CXCR3 antagonist as measured *in vivo* by fluorophotometry (n = 10 each). *(B,C,D)* Trabecular filtrating function is restored by treatment with CXCR3 antagonist as assessed by trabecular trapping of fluorescent microspheres (red), and quantitatively measured as percent of effective filtration length (PEFL), which is more important in treated eyes *(B)* than in untreated hypertensive eyes *(C)* (n = 10 each). *(E,F,G)* Density of apoptotic trabecular cells is lower in eyes treated with the CXCR3 antagonist *(E)* than in untreated hypertensive eyes *(F),* as assessed by TUNEL (green) and DAPI (blue) nuclear staining (n = 10 each). ** *P*<0.01. (scale bar, 50 µm). Data in bar graphs are presented as means ± SEM.

Investigations were conducted in the anterior segment of the eye in order to study mechanisms involved in the NBI-74330-related decrease in IOP. In our animal model, we observed a decrease in AH outflow together with a decrease in the TM filtrating surface in hypertensive eyes compared to normotensive controls, one month after the surgical procedure (**Fig. 5** ***A,B,C,D***). AH outflow impairment was significantly counteracted by treatment with NBI-74330 (**Fig. 5** ***A***). Furthermore, trabecular filtrating surface was also significantly improved by the selective CXCR3 antagonist (**Fig. 5** ***B,C,D***). TC apoptosis was significantly detected in hypertensive eyes as compared to normotensive controls (**Fig. 5** ***E,F,G***). In hypertensive eyes, apoptosis was significantly decreased by NBI-74330 as compared to untreated eyes. There was no inflammatory cell infiltration in the TM whatever the group as revealed by a lack of either anti-CD45 or anti-CD11b reactive cells. These data together suggested that blocking CXCR3 may lower OHT by restoring the trabecular filtrating function and protecting directly TCs from apoptosis.

### CXCR3 Antagonist Prevents Retinal Neuropathy and Protects Visual Function

**Figure 6:**
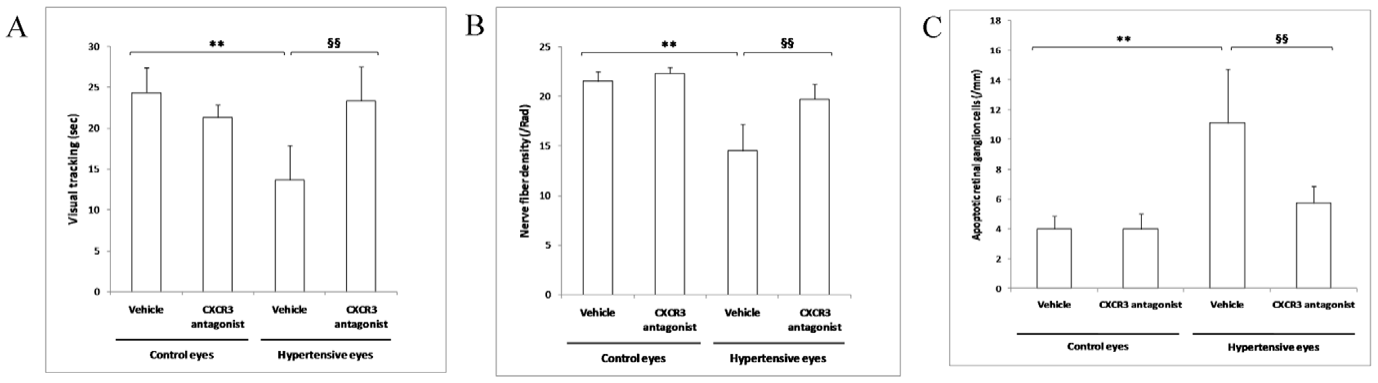
CXCR3 antagonism-induced lowering of intraocular pressure prevents retinal neurodegeneration and protects visual function in a rat model of ocular hypertension. *(A,B)* Ocular hypertension during 3 months is associated with a degradation in the visual function *(A)* as assessed by the duration of visual tracking during a 1-min optokinetic testing (spatial frequency, 0.5 cycle/degree), and with a decrease in retinal nerve fiber density *(B)* as measured *in vivo* by scanning light ophthalmoscopy. Ophthalmic treatment with CXCR3 antagonist significantly protects the visual function and prevents retinal nerve fiber loss (n = 10 each). *(C)* Ocular hypertension during one month is associated with an increase in retinal ganglion cell apoptosis (reported as the number of TUNEL-labeled cells normalized to the observed retinal layer length), which is reversed 15 days after the treatment with CXCR3 antagonist (n = 10 each). ** *P*<0.01 vs. normotensive eyes, §§ *P*<0.01 vs. untreated hypertensive eyes. Data in graphs are presented as means ± SEM.

In parallel, OHT-related retinal degeneration and related visual degradation were studied. In our animal model, retinal nerve fiber density was significantly decreased in eyes with OHT compared to controls three months after the surgery as assessed *in vivo* by confocal scanning laser ophthalmoscopy (**Fig. *6A***). Eyes treated with selective CXCR3 antagonist presented higher nerve fiber density than untreated hypertensive eyes (**Fig. *6A***). Similarly, the visual function was better in NBI-74330-treated eyes than in untreated controls as assessed by optokinetic measurements (**Fig. *6B***). *Ex vivo*, NBI-74330-related reduction in IOP was associated with a decrease in retinal ganglion cell apoptosis as assessed by TUNEL-labeling (**Fig. *6C***).

## Discussion

Several recent data have demonstrated that chemokines are not only involved in inflammatory/immune processes but also in the regulation of tissue microenvironment as well as in degenerative disorders [42]. In this study, we addressed the involvement of a balance between the full length chemokine CXCL12 and its truncated form SDF-1(5-67) in the regulation of IOP by modulating the TM filtrating function *via* the chemokine receptors CXCR4 and CXCR3. We describe the successful use *in vivo* of a non-peptide selective antagonist of CXCR3 to decrease elevated IOP and protect the retina in an animal model of OHT, establishing this chemokine receptor as a new therapeutic target to prevent the trabecular degeneration and its deleterious effects on the visual function.

We report that CXCL12, CXCR3, and CXCR4 are expressed in human trabecular tissue of glaucomatous patients as well as in a human glaucomatous TC line. In the eye, CXCL12 mRNA has been previously detected in TCs using gene microarrays [43]; CXCR4 has been investigated in the retina and in the cornea [19]–[21] but not in the TM; no data are available on the expression of CXCR3. TCs play a crucial role in IOP regulation because of their phagocytic activity and their ability to produce extracellular matrix components and MMPs [44], [45]. It has been recently demonstrated that MMP cleavage of CXCL12 yields a toxic truncated form, SDF-1(5-67), causing direct apoptosis *via* CXCR3 [27], [28]. MMPs have been reported to be significantly involved in mechanisms associated with TM degeneration in primary open-angle glaucoma [46]. Here we observed a basal production of SDF-1(5-67) by TCs, which originated from MMP activity on full length CXCL12. Interestingly, TNF-α and TGF-β2, cytokines known to be overexpressed in glaucoma [47], [48], increased the production of SDF-1(5-67). *In vitro*, we described a constitutive autocrine function of CXCL12/CXCR4 in TCs, as previously described in the brain [49], which protected TCs against apoptosis. Indeed, a direct protective role of full length CXCL12 has been reported in meningioma [50] and in a primary neuronal cell line [51] without any involvement of inflammatory cells. Recently, another chemokine receptor, CXCR7, has been identified as an additional receptor for CXCL12 [11]–[13]. In TCs, we observed a very low expression of CXCR7 which was not influenced by exposure to CXCL12; I-TAC/CXCL11, a reported ligand for CXCR7, was further unable to influence TC viability. In contrast, we observed that SDF-1(5-67) induced TC apoptosis through CXCR3 and activation of caspase-3. The involvement of CXCR3 in TC apoptosis was further confirmed by the use of NBI-74330 that inhibited the apoptotic effect of SDF-1(5-67), and by the use of IP-10/CXCL10, a cognate ligand for CXCR3, that mimicked its effect. However, the lack of effect of CXCL11 is intriguing since it is also one of the ligands for CXCR3. It could be explained, at least in part, by the fact that CXCL11 affinity is five-fold lower than that of CXCL10 for the spliced variant CXCR3-B, which is likely to be expressed by TCs [52]. Together our results suggest that SDF-1(5-67)-CXCR3 interaction induced TC loss through apoptotic mechanisms. Recently, Zhu *et al* reported that this interaction was also involved in autophagy suppression in neurons [53]. Though nothing is known about autophagic mechanisms involved in the trabecular degeneration, such a phenomenon cannot be excluded.

We thus decided to test selective chemokine receptor antagonists in a rat model of OHT in order to determine if CXCR3 and CXCR4 are involved in the trabecular pathology. Ophthalmic treatment with AMD-3100, a non-peptide selective antagonist of CXCR4, had no effect on IOP. On the contrary, NBI-74330, a non-peptide selective antagonist of CXCR3, induced a dose-dependent decrease in IOP and prevented retinal nerve fiber degeneration responsible for vision loss in hypertensive eyes. NBI-74330 has been described as a highly selective antagonist for CXCR3 in human and rodent cells [28], [29], [54], [55]. It was used *in vivo* to reduce atherosclerosis plaque formation in mice [56] and to slow growth of glioblastoma [57]. To our knowledge, this is the first study reporting the successful use of NBI-74330 for the treatment of an ocular disease.

We performed episcleral vein cauterization in rat eyes which leads to a stable OHT for months and to retinal degeneration as previously described [32], [58], [59]. While there is still a lack of accurate and validated animal models for glaucoma, we used this model because it shares several features with human primary open-angle glaucoma [60]: (i) it induces long-term OHT by reducing AH outflow without surgically damaging the TM; (ii) it does not modify the anterior segment anatomy [61]; and (iii) the sustained elevated IOP is associated with retinal nerve fiber defects and retinal ganglion cell apoptotic loss. In this animal model of OHT and OHT-related retinal degeneration, we report a decrease in the AH outflow together with a decrease in the trabecular filtrating function, suggesting that long-term OHT was due to late and stable trabecular dysfunction occurring after the initial vein obstruction. At the cellular level, we observed an increase in TC apoptosis similarly to what is observed in glaucoma in humans [10]. Ophthalmic treatment with NBI-74330 reduced IOP and related retinal degeneration by restoring the trabecular filtrating function and protecting TCs from apoptosis Even if the trabecular pathology in primary glaucoma is currently considered as a degenerative process, the relationship between glaucoma and inflammation has to be pointed out. It is noted that no inflammatory cell infiltration was found in rat TMs. Accordingly, our team previously found very few inflammatory cells in the TM of glaucomatous patients as assessed *ex vivo* by confocal microscopy [9]. Together these findings suggest that CXCL12 acts in autocrine/paracrine manner in the TM, and that glaucomatous trabecular degeneration is likely to develop without involving immune cells, further confirming that NBI-74330 acts directly on trabecular cells. Moreover, we reported *in vivo* that subconjunctival injections of SDF-1(5-67) in healthy rat eyes induced OHT. The transient effect of SDF-1(5-67) on IOP elevation may suggest that other factors can be implicated. Though our study focused on CXCL12, we cannot exclude that other chemokines could also play a role in the TM regulation, such as CXCL8, CCL2 and CXCL6 that were recently detected in cultured TCs, but whose effects are still unknown [62].

Here we originally suggest that the chemokine CXCL12 plays a crucial role in the initial pathogenesis of trabecular degeneration: (i) CXCL12 acts physiologically as a protective chemokine in an autocrine CXCL12/CXCR4 signaling mechanism; (ii) cytokine- and MMP-related pathological overexpression and processing of CXCL12 into SDF-1(5-67) decrease CXCL12-related CXCR4 activation and induce morphological changes and apoptosis *via* CXCR3, leading to TM dysfunction, OHT, and retinal degeneration; and (iii) CXCR3 blockade *in vivo* by ophthalmic treatment with a selective antagonist can restore trabecular function, prevent the retinal neuropathy and protect the visual function in an animal model of OHT. Current antiglaucoma treatment to decrease OHT is still non-curative because it does not treat the TM degeneration responsible for OHT that leads to retinal neurodegeneration and visual impairments. Here we propose that SDF-1(5-67)/CXCR3 interaction may be targeted in order to treat the causal trabecular pathology.

## Supporting Information

Figure S1 **CXCL12 and its truncated form SDF-1(5-67) bind to two different chemokine receptors.** Human glaucomatous trabecular cell line HTM3 was assessed for membrane expression of CXCR3 and CXCR4 using immunoflowcytometry. 3-h stimulation with exogenous CXCL12 (10 ng/mL [1.3 nM]) decreases membrane expression of CXCR4 but not CXCR3, whereas 3-h stimulation with SDF-1(5-67) (10 ng/mL [1.3 nM]) decreases the membrane expression of CXCR3 but not CXCR4; ** *P*<0.01. Data are presented as means ± SEM.
(DOCX)

Figure S2 **Lack of effect of a CXCR4 antagonist on intraocular pressure in a rat model of ocular hypertension.** Ophthalmic administration of a CXCR4 antagonist (AMD-3100, 1 µM, 100 µL) in the subconjunctival space does not modify intraocular pressure in control and surgically-induced hypertensive rat eyes (n = 10 in each group). Data are presented as means ± SEM.
(DOCX)

Figure S3 **SDF-1(5-67) increases intraocular pressure.** Two intraocular injections (black arrows) of exogenous SDF-1(5-67) (100 ng/mL [13 nM], 5 µL) in the anterior chamber of healthy rat eyes induce a transient ocular hypertension (n = 10 each); ** *P*<0.01 vs. vehicle-injected eyes. Data are presented as means ± SEM.
(DOCX)
