# Supplementary material for: CXCR3 Antagonism of SDF-1(5-67) Restores Trabecular Function and Prevents Retinal Neurodegeneration in a Rat Model of Ocular Hypertension
Source: PLoS One. 2012 Jun 4;7(6):e37873. doi: 10.1371/journal.pone.0037873 (PMC3366966; doi:10.1371/journal.pone.0037873)
Supplement: Figure S1 — CXCL12 and its truncated form SDF-1(5-67) bind to two different chemokine receptors. Human glaucomatous trabecular cell line HTM3 was assessed for membrane expression of CXCR3 and CXCR4 using immunoflowcytometry. 3-h stimulation with exogenous CXCL12 (10 ng/mL [1.3 nM]) decreases membrane expression of CXCR4 but not CXCR3, whereas 3-h stimulation with SDF-1(5-67) (10 ng/mL [1.3 nM]) decreases the membrane expression of CXCR3 but not CXCR4; ** P<0.01. Data are presented as means ± SEM. (DOCX) [file pone.0037873.s001.docx]

**Figure S1. CXCL12 and its truncated form SDF-1(5-67) bind to two different chemokine receptors.** Human glaucomatous trabecular cell line HTM3 was assessed for membrane expression of CXCR3 and CXCR4 using immunoflowcytometry. 3-h stimulation with exogenous CXCL12 (10 ng/mL [1.3 nM]) decreases membrane expression of CXCR4 but not CXCR3, whereas 3-h stimulation with SDF-1(5-67) (10 ng/mL [1.3 nM]) decreases the membrane expression of CXCR3 but not CXCR4; ** *P*<0.01. Data are presented as means ± SEM.
