# Supplementary material for: CXCR3 Antagonism of SDF-1(5-67) Restores Trabecular Function and Prevents Retinal Neurodegeneration in a Rat Model of Ocular Hypertension
Source: PLoS One. 2012 Jun 4;7(6):e37873. doi: 10.1371/journal.pone.0037873 (PMC3366966; doi:10.1371/journal.pone.0037873)
Supplement: Figure S2 — Lack of effect of a CXCR4 antagonist on intraocular pressure in a rat model of ocular hypertension. Ophthalmic administration of a CXCR4 antagonist (AMD-3100, 1 µM, 100 µL) in the subconjunctival space does not modify intraocular pressure in control and surgically-induced hypertensive rat eyes (n = 10 in each group). Data are presented as means ± SEM. (DOCX) [file pone.0037873.s002.docx]

**Figure S2. Lack of effect of a CXCR4 antagonist on intraocular pressure in a rat model of ocular hypertension.** Ophthalmic administration of a CXCR4 antagonist (AMD-3100, 1 µM, 100 µL) in the subconjunctival space does not modify intraocular pressure in control and surgically-induced hypertensive rat eyes (n=10 in each group). Data are presented as means ± SEM.
