# Supplementary material for: CXCR3 Antagonism of SDF-1(5-67) Restores Trabecular Function and Prevents Retinal Neurodegeneration in a Rat Model of Ocular Hypertension
Source: PLoS One. 2012 Jun 4;7(6):e37873. doi: 10.1371/journal.pone.0037873 (PMC3366966; doi:10.1371/journal.pone.0037873)
Supplement: Figure S3 — SDF-1(5-67) increases intraocular pressure. Two intraocular injections (black arrows) of exogenous SDF-1(5-67) (100 ng/mL [13 nM], 5 µL) in the anterior chamber of healthy rat eyes induce a transient ocular hypertension (n = 10 each); ** P<0.01 vs. vehicle-injected eyes. Data are presented as means ± SEM. (DOCX) [file pone.0037873.s003.docx]

**Figure S3. SDF-1(5-67) increases intraocular pressure.** Two intraocular injections (black arrows) of exogenous SDF-1(5-67) (100 ng/mL [13 nM], 5 µL) in the anterior chamber of healthy rat eyes induce a transient ocular hypertension (n=10 each); ** *P*<0.01 vs. vehicle-injected eyes. Data are presented as means ± SEM.
